# Community Compensatory Trend Prevails from Tropical to Temperate Forest

**DOI:** 10.1371/journal.pone.0038621

**Published:** 2012-06-11

**Authors:** Lin Xiao, Shixiao Yu, Mingguang Li, Yongfan Wang

**Affiliations:** State Key Laboratory of Biocontrol/School of Life Sciences, Sun Yat-sen University, Guangzhou, China; Michigan State University, United States of America

## Abstract

Community compensatory trend (CCT) is thought to facilitate persistence of rare species and thus stabilize species composition in tropical forests. However, whether CCT acts over broad geographical ranges is still in question. In this study, we tested for the presence of negative density dependence (NDD) and CCT in three forests along a tropical-temperate gradient. Inventory data were collected from forest communities located in three different latitudinal zones in China. Two widely used methods were used to test for NDD at the community level. The first method considered relationships between the relative abundance ratio and adult abundance. The second method emphasized the effect of adult abundance on abundance of established younger trees. Evidence for NDD acting on different growth forms was tested by using the first method, and the presence of CCT was tested by checking whether adult abundance of rare species affected that of established younger trees less than did abundance of common species. Both analyses indicated that NDD existed in seedling, sapling and pole stages in all three plant communities and that this effect increased with latitude. However, the extent of NDD varied among understory, midstory and canopy trees in the three communities along the gradient. Additionally, despite evidence of NDD for almost all common species, only a portion of rare species showed NDD, supporting the action of CCT in all three communities. So, we conclude that NDD and CCT prevail in the three recruitment stages of the tree communities studied; rare species achieve relative advantage through CCT and thus persist in these communities; CCT clearly facilitates newly established species and maintains tree diversity within communities across our latitudinal gradient.

## Introduction

Persistence of rare species in a community is intriguing. Compared with common species, rare species usually show low levels of self-compatibility, low overall reproductive effort and poor dispersal ability [1]. However, rare species still occur in nature and contribute a large proportion of the total number of species in most forests [2]–[6] (e.g. there are 300 rare species, accounting for 36% of all species of the Pasoh forest [6]). Thus, mechanisms that compensate for the disadvantages faced by rare species in competition with more common species could facilitate their persistence.

Some studies have postulated that compensatory mechanisms might be driven by density-dependent death rates [7]–[9]. These mechanisms could be general and have been termed ‘community compensatory trend (CCT)’ [4], [10]–[11]. Most researchers consider CCT as a process that confers advantage to rare species as a result of increased density dependent mortality with increasing abundance. Therefore, at the community level, a negative relationship between species population growth rates and abundance of the populations would indicate the existence of CCT.

**Figure 1:**
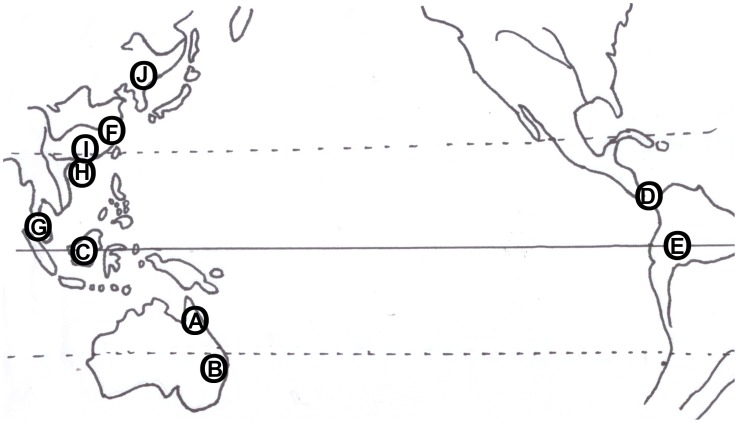
Study sites for investigating community compensatory trend (CCT). Locations supporting CCT: A) Davies Creek (DC), Australia [10]. B) Lamington National Park (LNP), Australia [10]. C) Gunung Palung National Park, Indonesia [4]. D) Barro Colorado Island (BCI), Panama [5], [12]. E) Yasuni National Park, Ecuador [13]. F) Gutianshan (GTS), China [14]. **Locations not supporting CCT:** D) Barro Colorado Island (BCI), Panama [15], [17]. E) Yasuni National Park, Ecuador [16]. G) Pasoh Reserve, Negeri Sembilan**,** Malaysia [6]. **Locations of our research:** H) Bawangling National Nature Reserve, China (‘Tropical’). I) Heishiding Nature Reserve, China (‘Subtropical’). J) Changbai Mountain National Nature Reserve, China (‘Temperate’).

In the past thirty years, there have been many attempts to document the action of CCT operating on the community level, but the results have been inconsistent**.** Some studies have shown the existence of CCT [4]–[5], [10], [12]–[14], while other do not find evidence of the process [6], [15]–[17] (Figure 1). Studies that support CCT found the per-adult recruitment rate declined with the increase of adult abundance [5], [10], [12]. In contrast, the studies that did not support CCT found significant positive relationships between seedling (or sapling) survival and the number of adults [6], [15]–[17]. In addition, a recent study [17] found that the effect of conspecific neighbors on survival was significantly and positively related to species abundance, with low-abundance species experiencing stronger NDD. These results question the occurrence of a CCT as a general mechanism promoting species coexistence.

The contrasting findings may result from use of different criteria for assessing populations growth rates. Even in the same forest, variation in criteria can lead to different conclusions. For example in BCI forest, Charles *et al.* [12] used per-adult recruitment rate to assess populations growth rates and their finding supported CCT; but Comita *et al*. used seedling survival for the assessment and their findings did not support CCT [15], [17]. This result may simply reflect that most low-abundance species possess higher per-adult recruitment rate and lower survival rate than do common species [5], [12]. However, survival is only one factor affecting species recruitment [5], [12]. It is worth noting that low survival rates of rare species can be offset by higher seed production or seedling survival [18] and be expressed as high recruitment per adult. Thus, at the community level, we argue that per-adult recruitment rate should be a better measure of population growth.

In addition, varied life stages and growth forms may also affect the assessment of CCT. For example, by considering growth forms, Connell *et al*. [10] found evidence for CCT only among understory trees. Thus, aspects such as the tree’s growth form and life stage should be considered while evaluating CCT.

Beyond the controversy about the general action of CCT, little is known whether CCT prevails across wide geographical ranges. Most studies addressing CCT have been conducted in the tropical forest [4]–[6], [10], [12]–[13], with few conducted in subtropical and temperate forests [14], [19]–[20]. Combining previous results, Lambers *et al.* [20] analyzed the relationship between the proportion of tree taxa experiencing density-dependent mortality and latitude. Their results indicated that density-dependent mortality did not increase over tropical latitudes, thus contradicting the idea that density-dependent mortality is responsible for the latitudinal gradient in species diversity. However, as an indirect assessment, this study may be insufficient to detail the relationship between NDD and latitude. For example, Lambers *et al.* did not consider that previous studies used two different criteria, based on, respectively, on tree life stage and growth, possibly leading to different inferences. Therefore, further studies are required to elucidate the relation between NDD and latitude.

In this study, we investigated the presence of CCT in tree communities at different latitudes and the relationship between NDD (or CCT) and latitude. We systematically established sample plots at three latitudes encompassing tropical, subtropical and temperate zones and analyzed the data with identical methods. We sought to detect NDD in all three communities and to compare NDD between rare and common species. Two widely used methods were applied to detect density dependence in these communities and distinguish the action of NDD between rare and common species.

## Methods

### Ethics Statement

Our observational and field studies were approved by the administration of the Bawangling National Nature Reserve, Heishiding Nature Reserve and Changbai Mountain National Nature Reserve, China.

### The Study Sites

#### Site 1

Bawangling National Nature Reserve (18°50’∼19°05’ N, 109°05’∼109°25’ E, Figure 1.H), Hainan Island, China (hereafter the ‘tropical’ site). The total area of this reserve is 72,000 hm^2^. The elevation of the study site is 800∼900 m, and the slope is 5∼15°. Climate is tropical monsoon, with an annual average temperature of 23.6°C, and annual precipitation of 1,500∼2,000 mm [21]. The vegetation is an old-growth tropical montane rain forest with *Dacrydium pierrei*, *Xanthophyllum hainanense*, *Syzygium araiocladum*, *Ixonanthes chinensis*, *Castanopsis hystrix* and *Syzygium chunianum* as the dominant species [22].

#### Site 2

Heishiding Nature Reserve (23° 25’∼23° 30’ N, 111° 49’∼111° 55’ E, Figure 1.I), Guangdong Province, China (hereafter the ‘subtropical’ site). This reserve has an area of 4,000 hm^2^. The study site at an elevation of 300∼400 m, with slopes of 15∼30°. Climate is seasonal, southern subtropical moist monsoon with annual average temperature of 19.6°C. The average annual precipitation is 1,743.8 mm, of which 79% falls between April and September. The vegetation is subtropical evergreen broadleaved forest [23], with *Cinnamomum porrectum*, *Cryptocarya concinna*, *Pinus massoniana*, *Quercus chungii* and *Schima superba* as the dominant species.

#### Site 3

Changbai Mountain National Nature Reserve (41° 42’∼42°1’ N, 127° 38’∼128° 0’ E, Figure 1.J), Southeastern Jilin Province, China (hereafter the ‘temperate’ site). The total area of the reserve is 196,465 hm^2^. The elevation of the study site is approximately 1,000 m with slope is less than 5°. The annual precipitation is 700∼1,400 mm and falls mainly during June, July and August. The annual average temperature is about 3∼7°C, and the relative humidity is 72∼75% [24]. The vegetation belongs to the Northeast China - Japan forest plant zone, including *Acer mandshuricum*, *Acer mono*, *Syringa reticulate* and *Tilia amurensis* as dominant species.

### Field Methodology

During 2007–2008, three 1-ha permanent plots (100 m×100 m) were established at each of the three study sites described above. Each plot was divided into 100 subplots (10 m×10 m). Four seedling plots (1 m×1 m) were placed around the center of every other subplot. In total, 600 seedling plots were located in 3-ha of permanent plots at each site (Figure S1).

All of the seedlings (0.1 cm≤ height <1.5 m, or diameter at breast height<1 cm) within the seedling plots were identified to species and enumerated. In the permanent plots, all of the individuals of height≥1.5 m were tagged, identified and enumerated, and their diameter at breast height (DBH; 1.3 m above the ground) measured. The tree species were assigned to a growth form based on architecture and maximum height attained: understory trees (4–10 m tall), midstory trees (10–20 m tall), and canopy trees (≥20 m tall) [10], [25]. In each growth form the trees with height≥1.5 m and DBH≥1 cm were categorized to three stages: sapling (1 cm<DBH≤2.5 cm); pole (2.5 cm<DBH≤5 cm for understory trees, 2.5 cm<DBH≤7.5 cm for midstory trees and 2.5 cm<DBH≤10 cm for canopy trees); and adult (DBH≥5 cm for understory trees, DBH≥7.5 cm for midstory trees and DBH≥10 cm for canopy trees).

### Analysis

#### Density dependent effect test

Two widely used methods were employed to test density dependent effects at the community level as follows.

The first one was used by Webb & Peart [4]. For all species, relative abundances were calculated as the ratio of species density in each size class to density of the same species in larger size classes. Under the assumption that species’ relative abundance does not change rapidly in time, a pattern of lower ratios in common species than in rare species is consistent with density-dependent mortality. Thus, this ratio was compared between common and rare species. If the relative abundance ratio in more abundant species is lower than in rare species density-dependent effects are strongly suggested (We used adult basal area as a proxy of abundance, following Webb & Peart, 1999, because basal area is a better indicator of tree biomass, canopy area and reproductive output than that of density alone [4]).

The second method has been used previously to detect density dependence for the transition from seed to seedling life stages [26], [27], to test whether abundance of reproductive adults negatively affects per capita number of seedlings [5], and to demonstrate rare species advantage in sapling recruitment [12].

We modeled relationships between seedling, sapling or pole abundance and adult abundance in three forest communities as:
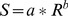
(1)This power function can be easily evaluated using least squares regression by, first, log transforming both sides of the equation:
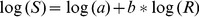
(2)where S is the number of established seedlings, sapling or poles, R is the number of adults, a is the mean density-independent seedling, sapling or poles establishment per adult, and b captures the effect of species abundance on established seedling, sapling or poles abundance. b = 1 indicates adults do not have density-dependent effect on the abundance of the focal stage (i.e., seedlings, sapling or poles); b<1 indicates a negative effect; b>1 indicates a positive effect.

#### Variation in density-dependence between different growth forms

Because we found the first method have better fitness than the second method under fewer data conditions, we analyzed variation in density-dependence between different growth forms exclusively using the first method.

#### CCT test

The existence of community-level density dependence alone might not provide any advantage to rare species. When a community experiences community-level density dependence, there are three possibilities: (1) all rare species show stronger density dependence than common species; thus, there is no rare species advantage; (2) all rare species show less conspecific density dependence than common species, and thus, a rare species advantage is said to occur; (3) the degree of density dependence relative to common species varies with rare species, and thus, overall rare species advantage will be determined by the proportion of rare species that show advantage. Using the log transformed power function, we estimated b for each species, thereby capturing the effect of species abundance on the abundance of seedlings, saplings or poles. We then modeled the relationship between b and log transformed adult abundance (adult basal area was also used as a proxy of abundance as before). If the proportion of b>1 for the less abundant tree species is larger than for most abundant species, we conclude that CCT exists.

## Results

### Individuals Enumerated and Data Used

At the Tropical Site, we counted 1,930 seedlings belonging to 115 species in the 600 seedling plots and 15,099 trees (DBH≥1 cm) belonging to 210 species in the 3-ha plot. At our Subtropical Site, there were 1,845 seedlings belonging to 82 species and 13,956 trees (DBH≥1 cm) belonging to 166 species. For our Temperate Site, we counted 2,698 seedlings belonging to 18 species and 5,968 trees (DBH≥1 cm) belonging to 44 species. The species numbers used for the analyses are shown in Table 1.

**Table 1:** Relationships between relative abundance ratio and adult abundance of three growth forms at different life stages in three forests.

Form	Stage	Location	N	Slope	95% CI for slope		R^2^	P
					Lower	Upper		
U	SD	TR	27	–0.315	–0.570	–0.035	0.2	0.0189
U	SD	STR	19	–0.350	–1.005	0.305	0.07	0.275
U	SD	TE	–	–	–	–	–	–
U	SP	TR	47	–0.073	–0.340	0.198	0.01	0.591
U	SP	STR	31	–0.320	–0.785	0.144	0.06	0.1689
U	SP	TE	–	–	–	–	–	–
U	PO	TR	45	–0.061	–0.269	0.148	0.01	0.5601
U	PO	STR	29	–0.433	–0.811	–0.054	0.17	0.0265
U	PO	TE	–	–	–	–	–	–
M	SD	TR	46	–0.374	–0.596	–0.152	0.21	0.0014
M	SD	STR	46	–0.416	–0.677	–0.154	0.19	0.0025
M	SD	TE	4	–0.922	–2.394	0.552	0.78	0.1147
M	SP	TR	56	–0.205	–0.365	–0.045	0.11	0.0132
M	SP	STR	55	–0.304	–0.556	–0.053	0.1	0.0185
M	SP	TE	6	–0.998	–2.951	0.954	0.34	0.2287
M	PO	TR	57	–0.137	–0.308	0.034	0.05	0.1134
M	PO	STR	60	–0.255	–0.439	–0.072	0.12	0.007
M	PO	TE	7	–0.412	–1.692	0.868	0.12	0.4455
C	SD	TR	28	–0.303	–0.771	0.165	0.06	0.1952
C	SD	STR	16	0.045	–0.708	0.797	0	0.9008
C	SD	TE	13	–0.422	–1.260	0.416	0.1	0.2913
C	SP	TR	32	–0.530	–0.834	–0.212	0.28	0.0018
C	SP	STR	19	–0.523	–0.916	–0.144	0.33	0.01
C	SP	TE	11	–0.762	–1.542	0.018	0.35	0.0546
C	PO	TR	30	–0.537	–0.781	–0.294	0.42	0.0001
C	PO	STR	20	–0.330	–0.690	0.031	0.17	0.0708
C	PO	TE	14	–0.530	–1.069	0.01	0.28	0.0536
Total	SD	TR	101	–0.178	–0.313	–0.043	0.06	0.0104
Total	SD	STR	81	–0.506	–0.664	–0.349	0.34	<0.0001
Total	SD	TE	17	–0.618	–1.063	–0.173	0.37	0.0098
Total	SP	TR	135	–0.195	–0.297	–0.093	0.1	0.002
Total	SP	STR	105	–0.379	–0.511	–0.247	0.24	<0.0001
Total	SP	TE	17	–0.920	–1.342	–0.498	0.59	0.0003
Total	PO	TR	132	–0.124	–0.214	–0.034	0.05	0.0073
Total	PO	STR	109	–0.243	–0.352	–0.134	0.15	<0.0001
Total	PO	TE	21	–0.555	–0.858	–0.253	0.44	0.0011

The upper of 95% CI of slope <0 indicates a significantly negative relationships. P<0.05 represents a signification relation. In our temperate study site there were only midstory and canopy growth form. U for ‘Understory’, M for ‘Midstory’, C for ‘canopy’, Total for all growth form, SD for ‘Seedling’, SP for ‘Sapling’, PO for ‘Pole’, TR for ‘Tropical’, STR for ‘Subtropical’, TE for ‘Temperate’, N for ‘Species number’.

### Density Dependence Test

**Figure 2:**
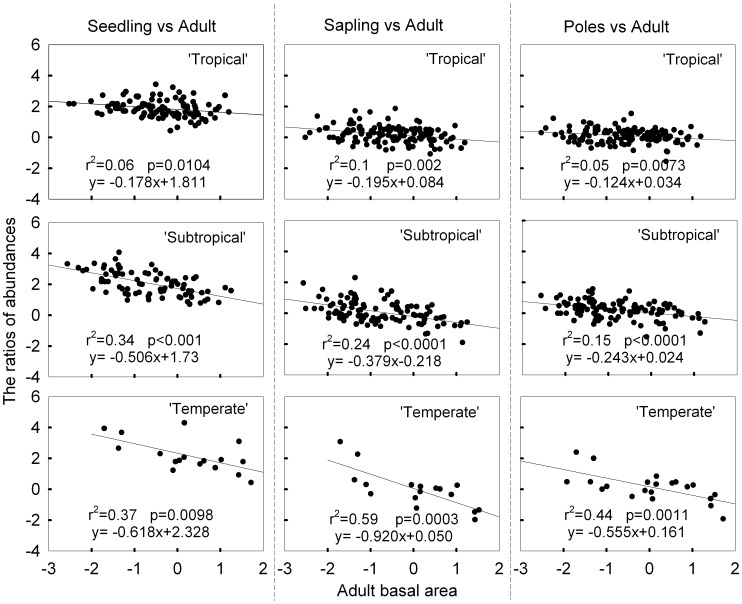
Relationship between the relative abundance ratio and species adult abundance in ‘Tropical’, ‘Subtropical’ and ‘Temperate’ study sites. Data have been log transformed. Species adult abundance was measured as adult basal area. The slope of the regression line significantly less than zero indicates relative abundance ratio declining with increasing adult abundance. Each dot represents one species.

The log relative abundance ratio was negatively correlated with log adult abundance across all three life stages (seedling, sapling and poles) in all three of the forest communities (Figure 2). For each life stage, the regression slope of the relative abundance ratio to adult abundance decreased with latitude (Table 1). Thus, according to these results from our first method, NDD exists in all three forest communities and the strength of NDD increases with latitude. Interestingly, the regression slope increased with tree size among the tree life stages in the subtropical forest community, but decreased a little for saplings in both tropical and temperate forest communities (Figure 2; Table 1). These results indicate that the effect of NDD varies among different life stages.

**Figure 3:**
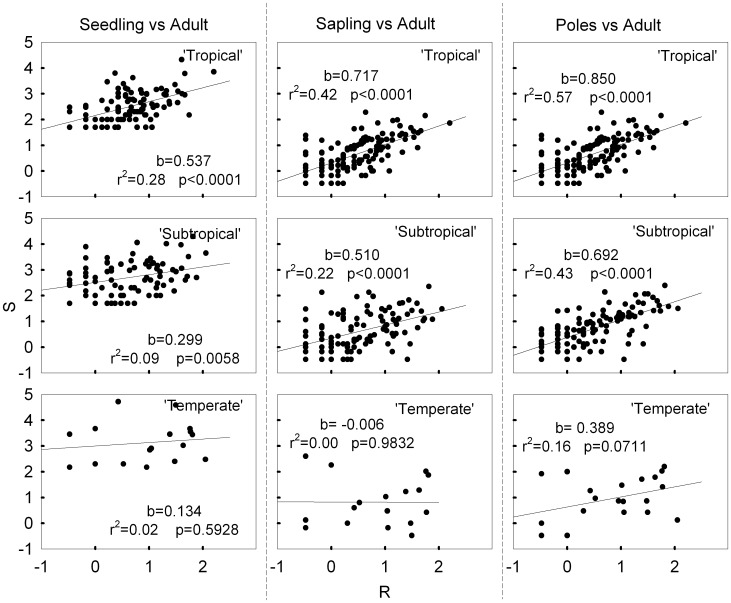
Relationship between the tree abundance at three life stages and adult abundance in three latitude communities. Note both axes are log transformed. The slope of the regression line is significantly less than one, indicating that per tree number of adult at each life stage declines with increasing adult abundance. Each dot represents one species.

The result of the power function analysis showed that the values of coefficient *b*, which captures the effect of species abundance on the establishment of seedlings, saplings or poles, are generally less than one in all three communities (Figure 3; Table 2). Therefore adult abundance had a negative effect on conspecific seedling, sapling and pole abundance across the latitudinal gradient. However, *b* decreased with latitude, indicating that the negative effect of adult abundance on seedling, sapling and pole abundance increased with latitude. The coefficient (*b*) increased with tree size in tropical and subtropical forest, but decreased for saplings in the temperate forest (Figure 3; Table 2). Thus, the degree of density dependence varied among life stages.

**Figure 4:**
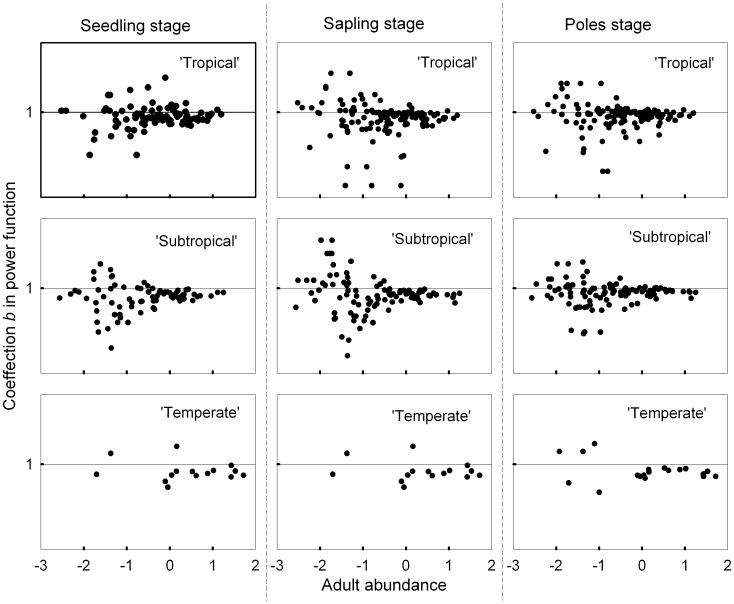
Relationship between each species’ power function coefficient b at three life stages and adult abundance in three latitude communities. Note X axes are log transformed. The dot above line *b* = 1, indicating that species have positive density dependence. The dot below line *b* = 1, indicating that species have NDD. Each dot represents one species.

**Table 2:** Relationships between seedling, sapling, poles abundance and adult abundance in three forest communities.

Stage	Location	N	*b*	95% CI for *b*		R^2^	P
				Lower	Upper		
SD	TR	101	0.537	0.365	0.768	0.28	<0.0001
SD	STR	81	0.299	0.089	0.509	0.09	0.0058
SD	TE	17	0.134	-0.389	0.658	0.02	0.5928
SP	TR	134	0.717	0.571	0.862	0.42	<0.0001
SP	STR	105	0.510	0.321	0.698	0.22	<0.0001
SP	TE	17	-0.006	-0.627	0.615	0.00	0.9832
PO	TR	132	0.850	0.721	0.980	0.57	<0.0001
PO	STR	109	0.692	0.539	0.846	0.43	<0.0001
PO	TE	21	0.389	0.106	0.692	0.16	0.0711

The upper of 95% CI of *b* <1 indicates a significantly negative relationships. P<0.05 represents a signification relation. SD for ‘Seedling’, SP for ‘Sapling’, PO for ‘Pole’, TR for ‘Tropical’, STR for ‘Subtropical’, TE for ‘Temperate’, N for ‘Species number’.

Different growth forms showed different patterns. The relative abundance of adult canopy tree species had significant negative relationships with saplings and poles in the tropical forest community, but only for saplings in the subtropical forest community. In contrast, the relative abundance of midstory tree species showed significant negative relationships between adult abundance and seedlings and saplings at the tropical site, but we found negative relationships for all life stages at the subtropical site. For understory trees, the negative relationship was significant for seedlings in tropical plots, but only for poles at the subtropical site (Table 1). This indicates that density dependent effects varied among growth forms and life stage.

### CCT Test

The relationship between power function coefficient *b* and adult abundance of species (Figure 4) showed that the coefficient for the more abundant trees was generally less than one. However, *b* values for less common species can fell into two groups, being either more or less than one. This pattern existed in all three latitudinal forest communities and did not obviously change with latitude (Figure 4). Thus, the fact that most common species exhibited NDD gave rare species a relative advantage and this pattern consistent with the CCT hypothesis prevailed from tropical to temperate forests existed.

## Discussion

### Wide-spread of Community-level Density Dependence

We have demonstrated that community-level density dependence is wide-spread in three forests along a latitudinal gradient in China. The relative abundance ratio had a negative relationship with adult abundance and the values of power function coefficient (*b*) were less than one for all three life stages (seedlings, saplings and poles) in all three communities. Therefore, our study not only confirms that NDD occurs in tropical [4]–[5], [10], [12]–[13] and subtropical forests [14], but also shows that NDD occurs in temperate forests. Clearly, this mechanism for generating rare species advantage is wide-spread across latitude.

Additionally, we found that the increase in density dependent with latitudes (i.e., the regression slope of relative abundance ratio to adult abundance, and the coefficient *b* decreases with latitude). As latitude increased, number of trees per unit area of forest decreased (for tree DBH≥1 cm, in the tropical forest community was 15,099 individuals/3ha, in the subtropical forest community was 13,956 individuals/3ha, and in the temperate forest community was 5,968 individuals/3ha). In contrast, however, species-specific density for adult trees increased with latitude, i.e., for adult trees densities were 0.0007, 0.0009, and 0.0020/m^2^, respectively, in the tropical, the subtropical and the temperate communities; for seedlings, the corresponding figures were 0.028, 0.038 and 0.250, respectively. Thus, the density ratios of seedlings to adults were 40.0, 42.2 and 125.0 at the tropical, the subtropical and the temperate site, respectively. These findings are consistent with the hypothesis that the probability that survivorship of a given species is negatively influenced by natural enemies or intraspecific competition increases with latitude, and that the shift is most notable between subtropical and temperate forest communities. Previous researches have shown that natural enemies and intraspecific competition both contribute to the NDD [7]–[8]; in addition, recent studies show that negative effects of pathogens can play a key role in NDD and that the impact of pathogens increase with increases in conspecific density [28]. Such relationships could contribute to the increase in NDD with latitude.

### NDD Varied among Different Growth Forms and Life Stages

Our results showed that the extent of NDD varied among both growth form and life stage in three forests (Table 1). Although canopy trees did not show NDD for seedlings in the tropical forest, they did show NDD at both sapling and pole stages, NDD was evident only at the sapling stage in the subtropical forest. Further, understory trees showed NDD at the seedling stage in tropical forest, in contrast, at the pole stage in subtropical forest.

These results partly confirm Connell’s hypothesis [10] that only understory trees show significant density dependence at the seedling stage in tropical forest. This might be reflect differences in life history strategy among tree growth forms in forests of different latitudes. In tropical forest, for example, canopy tree seedlings may demand less light while saplings and poles require more light [29]. Thus, the death rate of poles might raise for they could only reach to the midstory and their stronger light demands hardly be satisfied. In contrast, the poles of most subtropical canopy species might more easily reach to the top layer because most trees were already midstory species in this forest (Table 1).

Each tree growth form has specific light and other resource demands at specific life stages, and if these demands are unsatisfied, the tree may die. Since meeting these demands is affected by forest structure, something that changes with latitude and forest type, the same growth forms and tree species may show different degrees of NDD depending on forest and different latitude. Nonetheless, our research demonstrates the existence of NDD over a range of forest including typical tropical monsoon montane rain forest, subtropical and temperate broad-leaved forest. More studies are required, however, to determine the global extent of such effects in shaping forest vegetation.

### Rare Species Advantage

Our result show asymmetric density dependence: NDD operated for almost all common species, but data for only a portion of rare species gave evidence for such effects (Figure 4).This pattern can perhaps be explained in relation of the effects of microorganisms as follows. When a new species invades a community, it may accumulate microorganisms in the rhizosphere. For example, some rare tree species may accumulate more beneficial bacteria than pathogens [30], and their recruitment may be thus facilitated by and give rise to positive density dependence with respect to adult trees. Other rare species, however, may accumulate more pathogens than beneficial bacteria, and as a consequence, reflect a pattern of NDD. In contrast, most common species have been long present in their forest, providing ample opportunity for infection by soil pathogens [31]–[32]. Thus, they may more generally show higher density dependent mortality as adult abundance increases.

We considered this asymmetric density dependence operating across a community as CCT that contributes to a general rare species advantage. Thus, our argument differs from previous that either hold the existence of community-level NDD as CCT positive [7]–[9], or suggest that NDD is more common in rare than common species [17]. We argue that the existence of NDD can lead to three possible results. When all rare species show less NDD or the proportion of rare species showing NDD is less than for common species, community-level CCT may be entertained as a possible explanation for persistence of rare species. This situation was reflected in our results from 3 forest types, spanning a wide latitudinal gradient, and suggests that the hypothesis of CCT being driven by NDD has broad merit.

### NDD, CCT, Diversity Maintenance and the Latitudinal Gradient

We hold that NDD can promote maintenance of species diversity, and that this mechanism is wide-spread in forest communities. Our data corroborates previous researches [7]–[9] in suggesting that NDD can promote rare species advantage. In addition, we argue that CCT can also contribute to maintenance of forest tree diversity by contributing to rare species advantage.

We doubt that NDD and CCT contribute significantly to latitudinal gradient in tree diversity as previously proposed [33]–[34]. This is partially consistent with Lambers *et.al* [20] whose research showed that density-dependent mortality did not decrease with latitudes in tropics, and concluded that density-dependent mortality is not responsible for the latitudinal gradient in species diversity. However, our doubt raise from another reason: NDD increased rather dramatically from low to high latitude, and especially between our subtropical and temperate site; and CCT which is partly driven by NDD, did not show significant latitudinal differences.

### Conclusions

Our findings showed that CCT operates over a broad latitudinal range and that the advantage that a portion of rare species gain from CCT may allow them to persist in forest communities. This process clearly facilitates growth and propagation of newly established species and thus can contribute to maintaining species diversity in forest tree communities.

## Supporting Information

Figure S1 **Diagram of subplots and seedling plots in 1-ha permanent plot.**
(DOC)
