# Supplementary figures and images for: Community Compensatory Trend Prevails from Tropical to Temperate Forest
Source: PLoS One. 2012 Jun 11;7(6):e38621. doi: 10.1371/journal.pone.0038621 (PMC3372506; doi:10.1371/journal.pone.0038621)

**Figure S1** Diagram of subplots and seedling plots in 1-ha permanent plot.


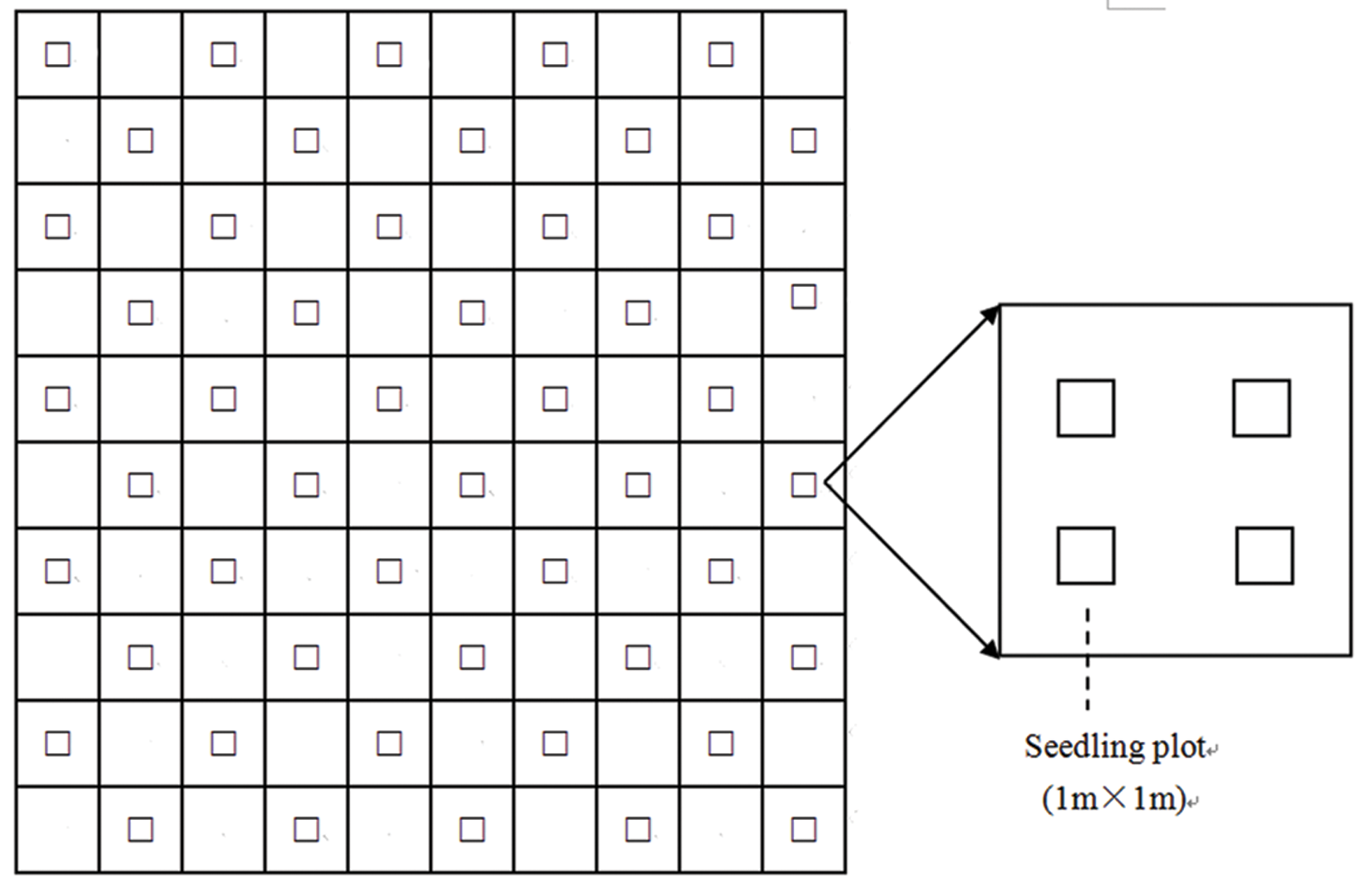

Supplement: Figure S1 — Diagram of subplots and seedling plots in 1-ha permanent plot. (DOC) [file pone.0038621.s001.doc]
